# Effects of Miniscalpel-Needle Release on Chronic Neck Pain: A Retrospective Analysis with 12-Month Follow-Up

**DOI:** 10.1371/journal.pone.0137033

**Published:** 2015-08-31

**Authors:** Shuming Li, Tong Shen, Yongshan Liang, Ying Zhang, Bo Bai

**Affiliations:** 1 Department of Rehabilitation Medicine, the First Affiliated Hospital of Guangzhou Medical University, Guangzhou, Guangdong, China; 2 Department of Orthopaedics Medicine, the First Affiliated Hospital of Guangzhou Medical University, Guangzhou, Guangdong, China; St Michael's Hospital, University of Toronto, CANADA

## Abstract

**Objective:**

Chronic neck pain is a highly prevalent condition, and is often treated with non-steroidal anti-inflammatory drugs. Limited clinical studies with short-term follow-up have shown promising efficacy of acupuncture as well as miniscalpel-needle (MSN) release. In this retrospective study, we examined whether MSN release could produce long-lasting relief in patients with chronic neck pain.

**Methods:**

We retrieved the medical records of all patients receiving weekly MSN release treatment for chronic neck pain at this institution during a period from May 2012 to December 2013. Only cases with the following information at prior to, and 1, 6, and 12 months after the treatment, were included in the analysis: neck disability index (NDI), numerical pain rating scale (NPRS), and active cervical range of motion (CROM). The primary analysis of interest is comparison of the 12-month measures with the baseline. Patients who took analgesic drugs or massage within 2 weeks prior to assessment were excluded from the analysis. For MSN release, tender points were identified manually by an experienced physician, and did not necessarily follow the traditional acupuncture system. MSN was inserted vertically (parallel to the spine) until breaking through resistance and patient reporting of distention, soreness or heaviness. The depth of the needling ranged from 10 to 50 mm. The release was carried out by moving the MSN up and down 3–5 times without rotation.

**Results:**

A total of 559 cases (patients receiving weekly MSN release treatment for chronic neck pain) were screened. The number of cases with complete information (NDI, NPRS, and CROM at baseline, 1, 6 and 12 months after last treatment) was 180. After excluding the cases with analgesic treatment or massage within 2 weeks of assessment (n = 53), a total of 127 cases were included in data analysis. The number of MSN release session was 7 (range: 4–11). At 12 months after the treatment, both NPRS and NDI were significantly lower [3 (0, 9) vs. 7 (5, 10) at the baseline for NPRS; [7 (0, 21) vs. 17 (9, 36) for NDI; p<0.001 for both]. All 6 measures of CROM were significantly higher at 12 months vs. the baseline. No severe complications (such as nerve damage and hematoma) were noted.

**Discussion:**

MSN release is effective, even 12 months after the treatment, in patients with chronic neck pain. Caution must be exercised in data interpretation due to the respective nature of the study and lack of a comparator group.

## Introduction

Chronic neck pain affects most adults at some point during their lifetime [1–3]. Annual prevalence of neck pain with no identifiable causes (non-specific neck pain) is estimated to be over 30% [4, 5].

Treatment for chronic neck pain includes exercise, massage, spinal manipulation, cervical traction, acupuncture, and the use of non-steroidal anti-inflammatory agents. Massage increases the compliance of soft tissue by mobilizing and elongating the connective tissue and shortening soft tissue [6–8]. A systemic review indicated that massage could provide immediate relief of neck and shoulder pain, but is not superior to acupuncture, exercise or traction [9]. Cervical manipulation could also relieve pain, increase cervical mobility, and minimize disability [10–13]. These conservative treatments are effective in the management of chronic neck pain, but most studies are limited by the short-term observation [10, 14].

Acupuncture has been shown to provide short-term benefits in patients with chronic neck pain [15–17]. Miniscalpel-needle (MSN) is similar to acupuncture needle, but has a flat-edged tip. MSN release is being increasingly used for a variety of pain conditions, including cervical myofascial pain syndrome, trigger thumb, plantar fasciitis, and joint pain [18–23].

Previous studies have reported superior efficacy of MSN release in patients with cervical myofascial pain syndrome over trigger point injection and acupuncture [18, 20, 23]. However, the follow-up in these studies was short-term. In the current retrospective study, we investigated whether MSN release could provide long-term benefits in patients with chronic neck pain.

## Methods

### Ethics Statement

This retrospective observational study was approved by the Ethics Committee of the First Affiliated Hospital of Guangzhou Medical University (Permit No 2015–15). Verbal informed consent for the MSN treatment was on medical records in all included cases. All patients signed informed consent to publish this material.

### Participants

All cases with non-specific chronic neck pain on electronic records in the Department of Rehabilitation Medicine of the First Affiliated Hospital of Guangzhou Medical University, during a period from May 2012 and December 2013 were reviewed. The diagnosis of non-specific chronic neck pain was established by attending physicians, and defined as pain of mechanical origin located in the neck, with or without radiation to the head, trunk or limbs [5]. All patients received either X-ray or magnetic resonance imaging to rule out possible spinal disorders.

### Intervention

**Fig 1:**
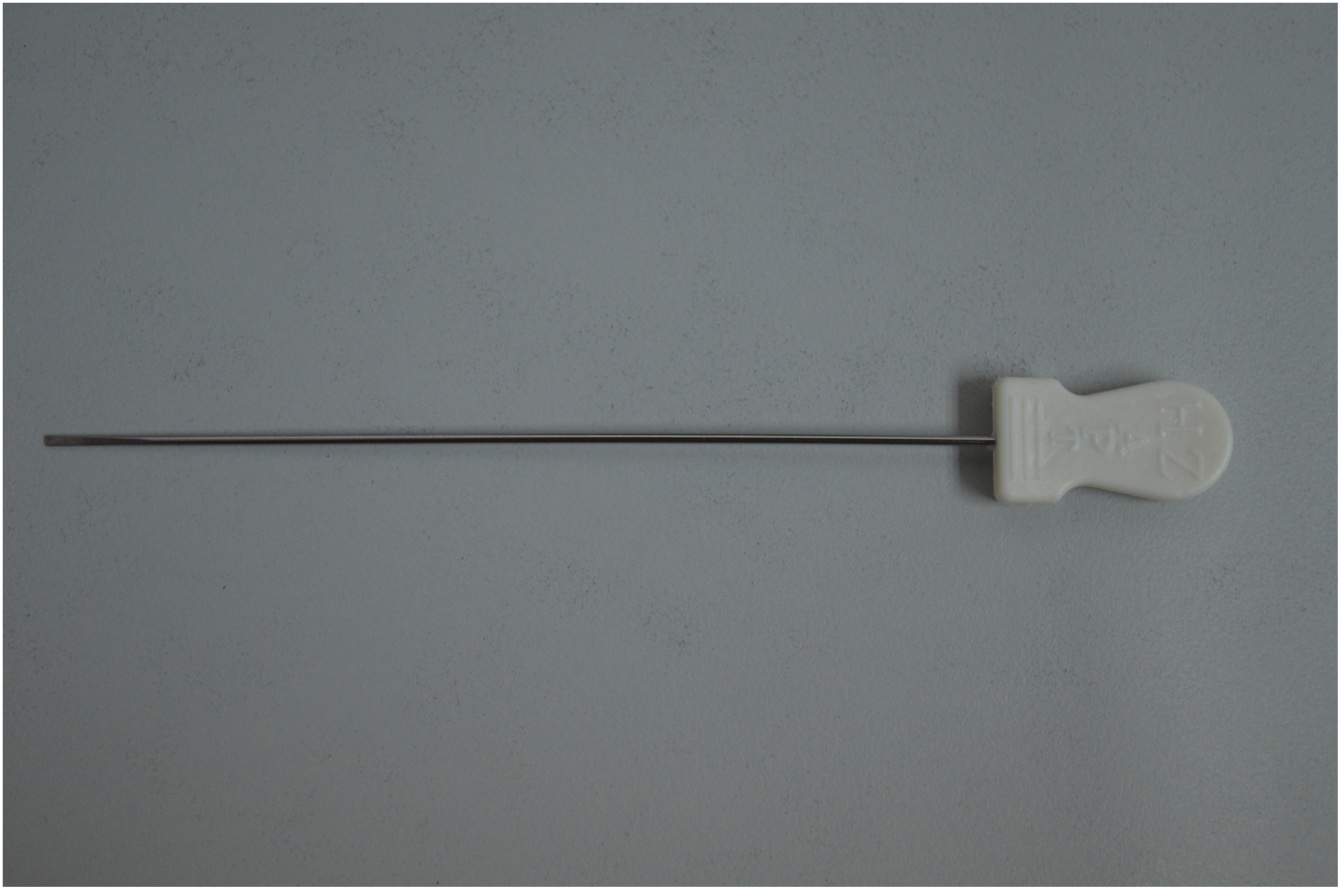
Photograph of a miniscalpel-needle (MSN).

**Fig 2:**
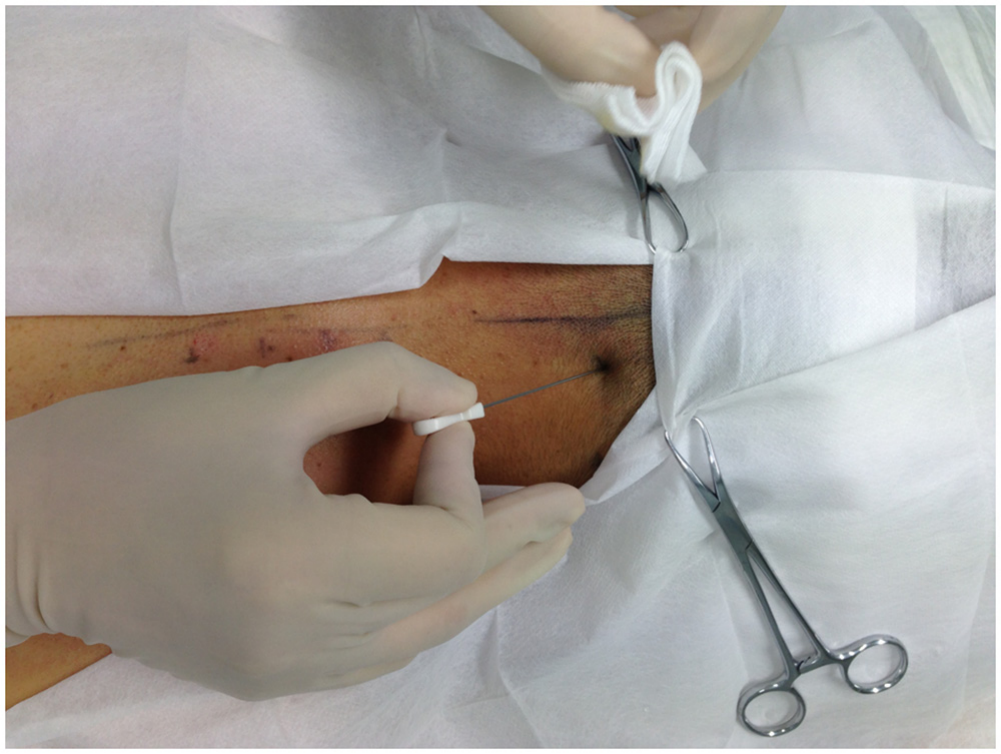
MSN release treatment.

The protocol for the MSN release treatment is based on previous clinical reports [18], and was performed in an out-patient operating room by an experienced physician (MSN specialist with 10 years of experiences). Patients sat in a chair with two arms resting on a couch. Painful tender points (Ashi points) were located by palpitation, and the area(s) was marked. After sterilization, the MSN (Fig 1, diameter 0.80 mm, length 60 mm, Huaxia Acupotomology Medical Equipment Factory, Beijing, China) was inserted vertically into the tender point, with the direction of the MSN parallel to the spine, until breaking resistance and patient reporting of distention, soreness or heaviness (Fig 2). The depth ranged from 10 to 50 mm. The release was performed by moving the MSN up and down 3–5 times without rotation. The MSN was subsequently withdrawn upon reduction of the resistance. Patients were monitored for 30 min after the MSN treatment. The treatment was repeated once a week for a duration deemed necessary by both the physician and the patients.

After the last session of the MSN treatment, patients received educational material containing: the use of hot packs, stretching of the upper trapezius muscle and levator scapulae muscle, and postural correction exercises. Patients were asked to return at 1, 6, 12 months for a follow-up examination.

### Outcome measures

The subjective outcome measures included pain intensity, as measured using a 10-point numerical pain rating scale (NPRS) [24] and neck pain-related dysfunction, as measured using the 10-item, 50-point neck disability index (NDI). The NDI included the following domains: subjective symptoms (pain intensity, headache, concentration, sleeping), daily activities (lifting, work, driving, recreation), and discretionary activities of daily living (personal care, reading) [25]. The Chinese version of NDI, previously validated [26], was used in the current study. The NDI was calculated using the following formula: (total score from 10 questions / 50) × 100%. For patients without driver’s licenses, the question on driving was excluded, in which case the NDI was calculated by the following formula: (total score from 9 questions / 45) × 100%.

Objective outcome measures were obtained using active cervical range of motion (CROM) [27]. The CROM instrument (Cervical Range of Motion Instrument, Orthopedic Physical Therapy Products, Minneapolis, USA) measures cervical range of motion for flexion, extension, lateral flexion, and rotation using three independent inclinometers attached to a frame: one inclinometer in the sagittal plane for flexion and extension, one in the frontal plane for lateral flexion, and one inclinometer in the transverse plane for rotation [28]. All patients were seated in a standard position during the examination. Three measurements were performed for each direction and the means were recorded.

### Statistical Analysis

Variables are reported as the mean ± standard deviation upon normal distribution, and as the median (range) otherwise. Data (NPRS, NDI and CROM scores) after the MSN release were compared to the baseline using the Wilcoxon matched-pairs signed rank test with SPSS Statistics 19 software (SPSS Inc, Chicago USA). Statistical significance was assumed if p < 0.05.

## Results

A total of 559 were identified in the screening. The number of cases with complete information (NDI, NPRS, and CROM at baseline, 1, 6 and 12 months after last treatment) was 180. After excluding the cases with analgesic treatment or massage within 2 weeks of assessment (n = 53), a total of 127 patients with chronic neck pain were included in data analysis (details in S1 Table).

The baseline characteristics of the patients, including age, sex, duration of symptoms, medication, NPRS, NDI, and CROM scores are listed in Table 1. The total number of MSN treatment sessions was 7 (range: 4–11).

**Table 1:** Baseline characteristics of the patients. MSN = miniscapel-needle; NPRS = Numerical Pain Rating Scale; NDI = Neck Disability Index; NSAIDs = non-steroidal anti-inflammatory drugs; CROM = active cervical range of motion.

	Patients with chronic neck pain
Age	49 (21, 60)
Gender ratio (M:F)	53:74
Duration of neck pain (months)	36 (12, 120)
NSAIDs	61
NPRS	7 (5, 10)
NDI (%)	17 (9, 36)
Flexion in CROM	35 (15, 45)
Extension in CROM	45 (20, 55)
Left side flexion in CROM	35 (15, 45)
Right side flexion in CROM	35 (15, 45)
Left rotation in CROM	50 (25, 60)
Right rotation in CROM	45 (30, 60)

### Subjective outcome measures

NPRS score was lower at 1, 6, and 12 months after the MSN release in comparison to the baseline (Table 2). The percentage of patients with at least 50% NPRS reduction was 80.3% at 1 month, 74.8% at 6 months, and 55.1% at 12 months. NDI also improved significantly (Table 2). The percentage of patients with at least 50% NDI improvement was 86.6% at 1 month, 80.3% at 6 months and 66.9% at 12 months.

**Table 2:** Effect of the MSN treatment on NPRS and NDI. MSN = miniscapel-needle; NPRS = Numerical Pain Rating Scale, NDI = Neck Disability Index.

	Baseline	1 month	6 months	12 months
NPRS	7 (5,10)	3 (0, 8) *	3 (0, 8) *	3 (0, 9) *
NDI	17 (9, 36)	5 (0, 20) *	6 (0, 20) *	7 (0, 21) *

* p<0.001 vs. the baseline.

### Objective outcome measures

All 6 measures of CROM increased significantly after the treatment when compared to the baseline (Table 3).

**Table 3:** Effect of the MSN treatment on all 6 measures of CROM. MSN = miniscapel-needle; CROM = active cervical range of motion.

	Baseline	1 month	6 months	12 months
Flexion in CROM	35 (15, 45)	45 (35, 55)*	45 (30, 55)*	45 (30, 55)*
Extension in CROM	45 (20, 55)	60 (45, 65)*	60 (40, 65)*	55 (35, 65)*
Left side flexion in CROM	35 (15, 45)	45 (35, 50)*	45 (30, 50)*	45 (30, 50)*
Right side flexion in CROM	35 (15, 45)	45 (35, 50)*	45 (30, 50)*	45 (30, 50)*
Left rotation in CROM	50 (25, 60)	65 (55, 70)*	65 (50, 70)*	65 (50, 70)*
Right rotation in CROM	45 (30, 60)	65 (55, 70)*	65 (50, 70)*	65 (50, 70)*

* p<0.001 vs. the baseline.

Sixteen patients (12.6%) experienced subcutaneous bleeding and transient distending pain; the symptoms/signs dissipated with management. No severe adverse complications (e.g., nerve damage and deep hematoma) were reported.

## Discussion

Previous studies have reported that MSN treatment produces greater short-term pain relief in patients with cervical myofascial pain syndrome than trigger point injection and acupuncture [18, 20, 23]. In a study with 3-month follow-up, Wang et al. reported that MSN treatment is superior to trigger point injection in both pain intensity and disability reduction in patients with cervical myofascial pain syndrome of the upper trapezius muscle [18]. Similarly, Ma et al. conducted a study with 3-month follow-up to compare MSN treatment with acupuncture needling and stretching exercises to trigger points in myofascial pain syndrome of the upper trapezius muscle, and found superior efficacy with MSN treatment [20]. Recently, Zheng et al. reported superior performance of ultrasound-guided MSN compared to ultrasound-guided dry needling therapy in reducing pain intensity and neck disability in patients with chronic neck pain after a 6-month follow-up [23].

In this study, we used both subjective (NPRS and NDI) and objective (CROM) outcome measurements to investigate the long-term effects of MSN treatment in patients with chronic neck pain. To minimize the impact of confounding factors, cases with the use of “pain-killers” or massage within 2 weeks prior to assessment were not included in the analysis. Such a design excluded majority of the cases. However, the results in the remaining cases (n = 127) were sufficient to show significant improvement at 12 months after the cessation of MSN release treatment in all 3 measures (NPRS, NDI and CROM).

MSN treatment was developed in China in the 1980s, and is structurally based on traditional acupuncture needles [29]. We believe that MSN treatment combines the therapeutic role of both acupuncture and micro-invasive operations [18, 20]. Specifically, detachment of the taut band by MSN in the tender points results in decreased muscle spasms, relaxation of compressed nerves and vessels, with overall improvements in the local microcirculation and restoration of the dynamic balance [30].

MSN treatment retains the benefits of traditional acupuncture needling. Numerous studies have shown that acupuncture could alter the metabolism of substrates involved in both the ascending facilitory pathway (N-methyl-D-aspartate receptors, substance P, and interleukin-1) and the descending inhibitory pain pathways (endogenous opioids, serotonin, and norepinephrine) [31–36]. For musculoskeletal conditions, the proposed mechanisms of acupuncture needling include micro-injury, increased local blood flow, facilitated healing, and analgesia [37]. A recent study provided new evidence of local molecular signaling in acupuncture analgesia by demonstrating that extracellular signal-regulated kinase (ERK) activation in the skin layer contributes to the analgesic effect of acupuncture in a mouse pain model [38].

Myofascial pain syndrome is one of the most frequent causes of chronic musculoskeletal pain [39, 40]. However, the pathophysiology of chronic neck pain remains largely unknown. Myofascial pain syndrome typically involves the trapezius muscle, levator scapula, sternocleidomastoid muscle, splenius capitis muscle, semispinalis capitis muscle, scalene muscle, and paraspinals [41]. Consistently, the tender points in the patients included in the current study were mostly at the above-mentioned muscles, suggesting that MSN treatment could relax the primary trigger points [20].

Potential adverse experiences of MSN release are typically minor, and include subcutaneous bleeding, coldness, burning sensation, and muscle pain [20]. In the current study, minor side effects of subcutaneous bleeding and transient distending were noted in 12.6% patients. No severe adverse experiences were noted during the entire 1-year follow-up, supporting the safety profile of MSN release.

The current study is retrospective by design. A randomized controlled trial with a comparator arm would have been more convincing, but practically difficult. The fact that majority of the initially screened subjects were not included in data analysis in the current study (a retrospective study) highlights the technical difficulty. Regardless, we attempted to minimize potentially important confounding factors. Specifically, patients who took analgesic agents or massage within 2 weeks of assessment were not included in data analysis.

## Conclusions

MSN release could provide long-lasting benefits in patients with chronic neck pain. The safety profile is acceptable.

## Supporting Information

S1 Table Demographics and clinical details of chronic neck pain: patient population. (XLS)
